# Lots of Narcissism Out There, Treatment Needed: Perspectives on Narcissism Among the General Public

**DOI:** 10.1002/pmh.70074

**Published:** 2026-04-10

**Authors:** David Kealy, Ellen F. Finch, Nicholas J. S. Day, John S. Ogrodniczuk

**Affiliations:** ^1^ Department of Psychiatry University of British Columbia Vancouver British Columbia Canada; ^2^ Department of Psychology Bates College Lewiston Maine USA; ^3^ School of Psychology University of Wollongong Wollongong New South Wales Australia

**Keywords:** narcissism, narcissistic, public opinion, stigma, survey

## Abstract

Narcissism is a hot topic in popular media, which often emphasizes harmful aspects and behaviours of individuals with narcissistic features. Such media likely contributes to negative perspectives that could further stigmatize narcissism and those who may suffer from narcissistic difficulties. However, limited research has investigated perspectives about narcissism among the general public. The present study surveyed 815 US‐based adults to explore public perspectives regarding the prevalence of narcissism, attitudes related to stigma, perspectives on suffering and hope, therapeutic issues and experiences of harm associated with narcissism. Findings indicate that a majority view narcissism as a prevalent and undesirable personality feature and warrant considerable clinical intervention. Individuals who endorsed having experienced harm by someone with narcissistic features were more likely to express negative perspectives. The mixture of public perspectives regarding narcissism seems to indicate a potential role for public education in order to reduce stigma and promote help‐seeking and treatment efforts.

Interest in narcissism and narcissistic pathology has surged in recent years, with an array of research studies and reviews contributing to ongoing academic debate about how to best understand these phenomena (Edershile and Wright 2022; Miller et al. 2021). Such interest is not confined to the research and clinical communities. Searching for ‘narcissism’ or ‘narcissistic’ on the Internet reveals a considerable volume of content, much of it apparently aimed at helping one recognize and deal with ‘the narcissist’ in one's life. Indeed, recent popular literature has emphasized the ill effects of narcissistic individuals on others, providing advice on how to detect and deal with ‘the narcissist’ in their workplace, family or other close relationships (e.g., Behary 2008; Hall 2019). Much of the self‐help industry surrounding narcissism seems to promote the message that individuals with narcissistic features are toxic, immune to psychological growth and deserving of disengagement. Indeed, self‐help advice offerings suggest that one not only avoid but neutralize or defeat the individual with narcissism (Dombek 2016). This media climate of fear and antipathy regarding narcissism—referred to by Dombek as the ‘narcissphere’—has been critiqued as contributing further confusion and stigma to narcissistic pathology (Dombek 2016). To refer to someone as ‘narcissistic’ today may well be done more as an insult rather than as a diagnosis for clinical targeting.

Indeed, the terms ‘narcissist’ and ‘narcissistic’ are often used pejoratively—perhaps more so than other psychiatric diagnoses—to denote a person who is almost irreparably self‐centred, unempathic and preoccupied with being admired or dominant. In reality, pathological narcissism is a spectrum of personality organized around compromised and maladaptive regulation of self‐image (Wright and Edershile 2018), with the diagnosis of narcissistic personality disorder (NPD) representing a particular and severe manifestation of this constellation (Weinberg and Ronningstam 2022). A vast theoretical and clinical literature speaks to the complexities involved in narcissistic pathology, particularly regarding its developmental dynamics and amelioration through psychotherapy. Clinicians often view the condition as ‘difficult to treat’ and requiring specialized interventions (Links and Prakash 2014; Luchner 2013). Moreover, research indicates that individuals with higher levels of pathological narcissism tend to have problematic interpersonal behaviours (Kealy et al. 2023; Roche et al. 2013), often contributing to emotional difficulties for their relatives and others (Day, Townsend, and Grenyer 2020).

Thus, one of the challenges for the field appears to be balancing and integrating such knowledge about narcissistic pathology—including its negative social impact—with the antipathy promoted in popular media. For example, echoing clinical conceptualizations (Gabbard and Crisp 2018; Caligor and Stern 2020; Kohut 1971), research has indicated pathological narcissism—especially narcissistic vulnerability—is associated with indicators of psychological suffering such as depression (Dawood and Pincus 2018), emotion dysregulation (Blay et al. 2024), loneliness (Finch and Kealy 2024) and increased substance misuse and risk‐taking (Kealy et al. 2017). Yet, popular discourse that equates narcissism with obnoxious selfishness may obscure awareness of and reduce sensitivity to such suffering. The ‘narcissist’ is often vilified in a moralistic sense and portrayed as impervious to personal growth and thus unlikely, if not unable, to change their hurtful ways (Dombek 2016).

These stigmatizing attitudes towards pathological narcissism might not only discourage individuals with the condition from seeking help but also marginalize them within mental health care systems. Further, although clinical experts note various potential challenges involved in the treatment of pathological narcissism (Gabbard and Crisp 2018; Weinberg and Ronningstam 2020), a climate of generalized stigma towards narcissism may infiltrate the perspectives of clinicians and contribute to undue pessimism (Penney et al. 2017). Indeed, mental health providers seem to regard narcissistic pathology as difficult to treat while also reporting limited training and experience in providing such care (Muir et al. 2021). Without direct and sustained experience in working with pathological narcissism, clinicians may be susceptible to echoing stigmatizing attitudes from the public sphere.

Previous research engaging non‐professionals regarding their understanding of narcissism and NPD has yielded somewhat mixed findings. A study among UK‐based community members found perceptions of ‘narcissists’ as superficial, disruptive and fragile—yet adept at leadership—with contradictory notions of treatability and treatment options (Wright and Furnham 2014). Moreover, in a separate study, these researchers found laypersons to be less likely to recommend psychological help for individuals with narcissistic features compared to those with depression or schizophrenia (Wright and Furnham 2015). A large online study of layperson's beliefs regarding narcissistic insecurity found majority endorsement of overt narcissism being related to an underlying sense of insecurity (Stanton et al. 2018). However, participants were not surveyed regarding compassion for narcissistic suffering, nor about implications for treatment. Participants in a study among German‐speaking respondents tended not to attribute grandiose narcissism to underlying vulnerability; ratings of likeability of people with narcissistic features were also unrelated to participants' theories of narcissism (Koepernik et al. 2022), although in a larger study vignettes illustrating combined grandiosity and vulnerability were rated more favourably than those with only grandiose narcissism (Villalongo Andino et al. 2023).

Collectively, extant research suggests a mixed picture regarding public perspectives on narcissism. Perceptions of narcissism as interpersonally difficult and less deserving of treatment—perhaps owing to public misinformation—seem to align with clinicians' perspectives on NPD as a highly stigmatized condition across general and healthcare contexts (Finch and Mellen 2025). Yet, the frequency of fear‐based stigmatizing attitudes among the public, or conversely, recognition of suffering remains unclear. Similarly, relatively little is known regarding public perspectives on the need for and responsiveness to treatment. Such knowledge is important because of the potential for clinicians to be biased by and responsive to popular attitudes. Thus, the present study was developed as an effort to further investigate several questions related to perspectives on narcissism in the general population.

How prevalent is narcissism perceived to be in the broader community and in people's own lives? To what extent do people harbour fearful and stigmatizing attitudes towards individuals with narcissistic difficulties? And how are issues such as suffering, harm and treatment regarded? The present study used a survey approach to examine these questions, exploring perceptions of individuals described as ‘narcissistic’ rather than specific diagnostic criteria or clinically derived vignettes.

## 1 Methods

### 1.1 Participants and Procedures

The present study is part of a larger project on public impressions and experiences of mental health. The study was reviewed and approved by the Behavioural Research Ethics Board of the University of British Columbia, and the procedures followed were in accordance with the Helsinki Declaration as revised in 2013. The patients/participants provided informed consent to participate in this study. A nationally representative sample of US‐based participants was sourced through the Prolific (https://www.prolific.com/) recruitment platform. Participants were adults who responded to advertisements about a research survey regarding perspectives on personality, relationships and psychological treatment. Information about the study was provided, and prospective participants were invited to consent to take part in the study by initiating questionnaire completion. A modest payment of $4 was offered to participants. A total of 837 participant entries were obtained. Review of data quality resulted in the removal of several entries due to failure to complete the survey (*n* = 14), duplicate responses (*n* = 4) and inattentiveness based on minimal response time (*n* = 4). Although several participants failed one (*n* = 41) or two (*n* = 2) out of four attention checks throughout the survey, no participants failed three or more of these items, and hence, no data were removed on this basis. Following these data screening procedures, a final sample of *N* = 815 was obtained.

Participants' average age was 45.6 years (SD = 15.83; range = 18–86), reported by 805 respondents; 10 participants did not indicate their age. Respondents' gender identity included 49.8% (*n* = 406) identifying as female, 48.3% (*n* = 394) identifying as male and 1.7% (*n* = 15) identifying as non‐binary or another gender identity. Sexual orientation was indicated as 78.9% (*n* = 643) as heterosexual, 10.2% (*n* = 83) as bisexual, 4.5% (*n* = 37) as gay or lesbian and 2.6% (*n* = 21) as asexual, with 3.6% (*n* = 30) indicating other sexualities. Regarding cultural background, 63.7% (*n* = 519) identified as European, 13.6% (*n* = 112) as Hispanic or Latino/Latina, 12.9% (*n* = 105) as African, 7.4% (*n* = 60) as Asian, 4.2% (*n* = 34) as Indigenous, 2.2% (*n* = 19) as Middle Eastern and 1.4% (*n* = 11) as South Asian; 7.5% (*n* = 61) indicated other cultural backgrounds (participants could indicate multiple responses). Most participants were educated beyond high school, including 59.1% (*n* = 482) with a university degree, and employed either full‐time, 50.4% (*n* = 411), or part‐time, 16% (*n* = 130). The majority, 62.3% (*n* = 508), reported being in committed partner relationships, with 37.3% (*n* = 304) single or dating casually.

### 1.2 Materials

The survey included in the present study consisted of 12 statements regarding perceptions of narcissism. Rather than describe the clinical criteria for NPD or dimensionality of pathological narcissism, we developed survey items using language common to lay contexts, such as ‘narcissistic person’ and ‘people who are narcissistic’. Items were developed to cover (1) perceptions of prevalence and presence of people with narcissistic features; (2) respondents' stigma‐related attitudes regarding narcissism; (3) perspectives on the degree of suffering and prospects for change associated with narcissism; (4) opinions about therapeutic effectiveness in ameliorating narcissism; and (5) direct negative experience with an individual perceived as being narcissistic. Each item was scored using a Likert scale ranging from 1 (*strongly disagree*) to 5 (*strongly agree*). The items are displayed in the reporting of results (Table 1).

**TABLE 1:** Descriptive statistics for responses to survey items regarding perceptions of narcissism.

		Item response					
	*N*	1	2	3	4	5	*M* (SD)
There seem to be a lot of narcissistic people in the world	814	14 (1.7%)	78 (9.6%)	163 (20%)	410 (50.3%)	149 (18.3%)	3.74 (0.92)
I seem to have a lot of narcissistic people in my life	811	100 (12.3%)	341 (41.8%)	187 (22.9%)	141 (17.3%)	42 (5.2%)	2.61 (1.07)
I have a fear of narcissistic people	813	153 (18.8%)	266 (32.6%)	165 (20.2%)	161 (19.8%)	68 (8.3%)	2.66 (1.23)
People who are narcissistic do not deserve to do well in life	812	128 (15.7%)	215 (26.4%)	325 (39.9%)	105 (12.9%)	39 (4.8%)	2.65 (1.05)
I would hate it if someone thought I was narcissistic	814	52 (6.4%)	58 (7.1%)	131 (16.1%)	308 (37.8%)	265 (32.5%)	3.83 (1.15)
People who are narcissistic are truly suffering	814	47 (5.8%)	166 (20.4%)	323 (39.6%)	218 (26.7%)	60 (7.4%)	3.10 (1.00)
People who are narcissistic deserve help	815	18 (2.2%)	29 (3.6%)	179 (22%)	401 (49.2%)	188 (23.1%)	3.87 (0.88)
People who are narcissistic cannot really be helped	815	156 (19.1%)	325 (39.9%)	212 (26%)	90 (11%)	32 (3.9%)	2.41 (1.04)
People who are narcissistic need a lot of therapy	813	13 (1.6%)	30 (3.7%)	233 (28.6%)	267 (32.8%)	270 (33.1%)	3.92 (0.95)
Psychotherapy is effective for people who are narcissistic	815	37 (4.5%)	71 (8.7%)	403 (49.4%)	239 (29.3%)	65 (8%)	3.27 (0.90)
People who are narcissistic would be difficult for most therapists to treat	815	49 (6%)	134 (16.4%)	232 (28.5%)	283 (34.7%)	117 (14.4%)	3.35 (1.10)
I have been hurt by a narcissistic person in the past	814	92 (11.3%)	155 (19%)	80 (9.8%)	270 (33.1%)	217 (26.6%)	3.45 (1.36)

*Note:* 1 = *strongly disagree*; 2 = *disagree*; 3 = *neither disagree nor agree*; 4 = *agree*; 5 = *strongly agree*.

### 1.3 Analyses

Analyses were conducted using SPSS 30. Responses to survey questions were examined descriptively and with exploratory tests regarding patterns based on gender, age and endorsement of having ever been hurt by a narcissistic individual. Descriptive statistics were computed to indicate participants' responses to survey items. Inferential statistics were computed using non‐parametric tests due to the ordinal nature of item response options and non‐normal distributions across various items. Missing responses were minimal (< 1%) and were omitted from analyses on a test‐by‐test basis. Response patterns based on gender (male, female and non‐binary/other) and age (continuous) were examined using independent samples Kruskal–Wallis tests and Spearman correlations, respectively. Spearman correlations were also used to explore response patterns based on endorsement of having been hurt by a narcissistic individual; the responses to this item were examined in relation to the remaining items. Statistical significance was set at *p* < 0.01 for all analyses due to multiple significance tests; only significant associations are reported in Section 2.

## 2 Results

Descriptive statistics for survey item responses are displayed in Table 1. As indicated by descriptive statistics, the average responses across all items were between 2 (*agree*) and 4 (*disagree*).

### 2.1 Perceptions of Prevalence

A majority of respondents perceived narcissism to be prevalent in the community, with two‐thirds (68.6%) agreeing with the statement, ‘there seem to be a lot of narcissistic people in the world’. Only 11.4% indicated disagreement with this perception. However, considerably fewer people felt there was a large prevalence of narcissism in their own social surround, as more than half (54.1%) disagreed with the item, ‘I seem to have a lot of narcissistic people in my life’. Nevertheless, nearly one in four respondents (23.5%) endorsed being around many individuals with narcissistic features. These respondents tended to be younger, indicated by a modest negative association between agreement with this item and age, *r*_*s*_(799) = −0.15, *p* < 0.001.

### 2.2 Stigma‐Related Attitudes

Items regarding stigma‐related attitudes yielded a mixture of responses. The item addressing ‘fear of narcissistic people’ was endorsed by a minority, 28.1%, of respondents. Female participants were more likely than males to endorse such fear, *H* = 22.3, *p* < 0.001, as were younger participants, *r*_*s*_(802) = −0.13, *p* < 0.001. However, half the sample, 51.4%, denied having any such concern. Similarly, a relatively small minority expressed antipathy towards individuals with narcissistic features, with only 17.7% indicating that ‘people who are narcissistic don't deserve to do well in life’. More than twice that number of respondents, 42.1%, indicated their disagreement with this attitude. Yet, when presented with the notion of being deemed narcissistic by someone else, most respondents affirmed their discomfort: 70.3% agreed—and only 13.5% disagreed—with the statement, ‘I would hate it if someone thought I was narcissistic’. Agreement with this item tended to be stronger among female compared to male participants, *H* = 38.17, *p* < 0.001.

### 2.3 Perspectives on Suffering and Hope

One third of respondents, 34.1%, endorsed the notion that ‘people who are narcissistic are truly suffering’. However, one in four (26.2%) disavowed that sentiment. Nevertheless, a large majority, 72.3%, indicated that ‘people who are narcissistic deserve help’. Female respondents (compared to males) tended to give stronger endorsement to this item, *H* = 16.42, *p* < 0.001. Only 5.8% felt that individuals with narcissism do not deserve help. A majority of respondents also refuted a pessimistic outlook regarding the potential for change in narcissism, with 59% disagreeing with the statement, ‘people who are narcissistic cannot really be helped’, and only 14.9% endorsing this perspective.

### 2.4 Therapeutic Issues

As with opinions about individuals with narcissism deserving and potentially benefitting from help, perceptions of potential therapeutic needs were endorsed by a majority of respondents. Nearly two‐thirds, 65.9%, agreed—and only 5.3% disagreed—with the statement, ‘people who are narcissistic need a lot of therapy’; endorsement of this item was stronger among female compared to male participants, *H* = 35.17, *p* < 0.001. Fewer respondents, however, were certain of the effectiveness of treatment, with 37.3% supporting the notion that ‘psychotherapy is effective for people who are narcissistic’. Whereas nearly half the sample gave a neutral response to this item, a minority (13.2%) indicated a negative outlook regarding therapeutic efficacy. Many respondents also perceived narcissism to be a challenging issue to address in therapy, with 49.1% agreeing that ‘people who are narcissistic would be difficult for most therapists to treat’. This statement was endorsed more strongly by female compared to male respondents, *H* = 15.16, *p* < 0.001. Nevertheless, one in four (22.4%) respondents disagreed, indicating confidence that narcissism could be managed by therapists with little difficulty.

### 2.5 Experience of Harm

One survey item referred to personal experience of having been harmed through interaction with an individual with narcissistic features. More than half the sample, 59.7%, indicated some agreement with the statement, ‘I have been hurt by a narcissistic person in the past’. Endorsement of this item was stronger among female and non‐binary compared to male participants, *H* = 49.5, *p* < 0.001, and was modestly negatively associated with age, *r*_*s*_(802) = −0.12, *p* < 0.001. However, 40.1% were either neutral or indicated having never experienced harm in the context of others' narcissism.

Respondents' perceived personal harm from narcissistic interaction was examined in relation to the aforementioned survey items. Those who endorsed having experienced such harm were more likely to perceive narcissism as prevalent in the community, *r*_*s*_(811) = 0.32, *p* < 0.001, and in one's life, *r*_*s*_(808) = 0.36, *p* < 0.001. The experience of harm was also positively associated with fear of narcissism, *r*_*s*_(810) = 0.38, *p* < 0.001, antipathy towards individuals with narcissism, *r*_*s*_(809) = 0.19, *p* < 0.001, and personal discomfort at being seen as narcissistic, *r*_*s*_(811) = 0.28, *p* < 0.001. Modest associations were also observed with recognition of suffering in narcissism, *r*_*s*_(811) = 0.11, *p* = 0.003, and deservedness of help *r*_*s*_(812) = 0.11, *p* = 0.002, yet also with pessimism about such help, *r*_*s*_(812) = 0.16, *p* < 0.001. Although experience of harm was unrelated to opinions about the effectiveness of therapy for narcissistic problems, endorsement of harm was moderately associated with a perceived need for extensive treatment *r*_*s*_(810) = 0.27, *p* < 0.001, and anticipated therapist challenges, *r*_*s*_(812) = 0.27, *p* < 0.001.

## 3 Discussion

Despite an apparent explosion of fascination with narcissism in both academia and popular media, there have been limited data regarding the public's perception of narcissism. Findings from this survey of American adults indicate that a large majority sees narcissism as a prevalent personality feature. A similar proportion seems to view narcissism as an undesirable characteristic, preferring not to be regarded as narcissistic by others. However, a relatively smaller share of the sample endorsed fear or condemnation of narcissism. Indeed, a majority of the public seems to view narcissism as a condition deserving of and benefitting from a considerable amount of clinical intervention. Perhaps not surprisingly, more negative and pessimistic opinions about narcissism were expressed by individuals—more than half the sample—who endorsed having been hurt by someone they perceived as having narcissistic features. Taken together, findings indicate a mixed picture of public perceptions, indicating a potential role for public education regarding the nature of narcissistic personality functioning.

The perception that ‘a lot’ of people in society have narcissistic features, which was endorsed by the majority of the sample, suggests a discrepancy with relatively low empirical estimates of the prevalence of NPD. Population studies have suggested incidence of NPD ranging from less than 1% to nearly 6% (Dhawan et al. 2010). It is possible that public perception of a high prevalence of narcissistic dysfunction has been shaped by popular and self‐help media, including messages that imply narcissism as a root cause of various offensive interpersonal interactions. By labelling an expansive set of interpersonal difficulties ‘narcissistic’, these messages may inflate perceptions of prevalence: If an ever‐wider swath of antagonistic behaviours are assumed by the public to be diagnostic markers, then many alleged ‘narcissists’ will be apparent. This finding may also reflect the controversial but widely publicized claim that narcissism has been on the rise over time. Despite limited evidence (Hamamura et al. 2020; Wetzel et al. 2017), authors have raised alarm over the prominence of narcissism (Twenge and Campbell 2009), with recent emphasis on excessive social media and ‘screen culture’ as outlets for narcissistic self‐enhancement (Casale and Banchi 2020). However, the Diagnostic and Statistical Manual version of NPD has long been critiqued for failing to capture the range of narcissistic dysfunction (Levy et al. 2013; Schalkwijk et al. 2021), with recent emphasis directed towards pathological narcissism as a dimensional constellation of self‐image dysregulation. Socially and/or clinically relevant pathological narcissism is likely much more prevalent than NPD, and this may be reflected to some extent in responses to our survey.

That more than half of respondents associated personal harm with an individual they believed to exhibit narcissistic features—with such experience being associated with more negative perceptions of narcissism overall—tallies with research documenting significant burden among relatives of people with pathological narcissism (Day, Bourke, et al. 2020). Maladaptive patterns of interaction, including tendencies towards dominance, vindictiveness and coldness, tend to accompany higher levels of pathological narcissism (Kealy and Ogrodniczuk 2011). Indeed, partners and family members of narcissistic individuals report experiencing their relative as controlling and sometimes abusive (Day et al. 2022). Studies have linked narcissistic features with unresponsive parenting (Hart et al. 2017), impaired capacity for intimacy (Finch and Kealy 2024) and angry and aggressive responses towards others (Krizan and Johar 2015). Together with our respondents' experiences of the negative impact of narcissism, such findings underscore the considerable interpersonal aspect of personality dysfunction (Wright et al. 2022). However, people who have experienced interpersonal harm from individuals with narcissistic features may, in search of support, turn to online sources that promulgate concepts of ‘narcissistic abuse’, further entrenching the notion of unique dangers being associated with such individuals.

Despite perceptions regarding its prevalence and negative impact, fear and moral condemnation of narcissism do not appear to be especially prominent in the general public. Less than 5% of respondents strongly agreed that narcissistic individuals do not deserve to succeed in life. Moreover, although the majority were unconvinced of the suffering involved, one‐third of respondents believed narcissism to be a condition with significant suffering for the individual and not simply a source of aggravation for others. The vast majority also felt that narcissism is deserving of clinical attention. Further public mental health communications could leverage these opinions—and provide education about the impairment and associated suffering—in order to reduce stigma regarding pathological narcissism and build public support for increasing treatment availability. Some research suggests that recognition of vulnerability, compared to seeing only grandiosity, within narcissism is associated with more sympathetic attitudes towards individuals with narcissistic features (Villalongo Andino et al. 2023).

Stigma remains a conspicuous obstacle to improving access to and effectiveness of psychological help for individuals with pathological narcissism. Indeed, most of our respondents would recoil at someone else considering them to have narcissistic features. Even among mental health professionals, terms like ‘narcissistic’ are often used disparagingly rather than to signify a target for intervention (Penney et al. 2017). Qualitative research has highlighted stigma at individual, social and structural levels as a critical barrier to effective diagnosis and treatment for individuals with pathological narcissism (Finch and Mellen 2025). Indeed, responses to our survey underscore the need for sensitivity among clinicians when introducing narcissism into conversations with patients (Hersh et al. 2019). The public's relatively limited antipathy towards and emerging recognition of suffering among individuals with narcissism may indicate a pathway for compassionate discussion of narcissistic dynamics in clinical encounters. Yet, an aversion to being labelled ‘narcissistic’ highlights a need for caution and care—perhaps with an emphasis on the personal suffering involved in narcissistic difficulties—when doing so. Although the public seems aware of the need for serious treatment options—with two‐thirds of respondents indicating ‘a lot’ of therapy is required to treat narcissistic difficulties—opinions regarding the effectiveness of therapy are mixed. To some extent, these opinions may reflect the lack of availability of empirically supported treatment for pathological narcissism (Dhawan et al. 2010), disparate approaches advocated by clinicians (Kealy et al. 2017) and clinical lore that suggests narcissism is difficult to treat (Finch and Mellen 2025). Specific treatment methods have been articulated for NPD and pathological narcissism (Caligor et al. 2019; Drozek and Unruh 2020), yet there is a need for rigorous research evaluating the efficacy of these and other potentially suitable approaches. Such research, along with education and training about treating narcissistic difficulties, may help to shift public and clinician perspectives and further reduce stigma.

Several limitations to the present study must be mentioned. First, although sampling was intended to demographically reflect the US population, it is likely that the perceptions and opinions we sampled could vary depending on demographic, political and social characteristics that were not accounted for. It is possible, for example, that respondents' understanding of narcissism‐related terminology may differ on such grounds. Indeed, our use of informal and non‐operationalized terminology invites caution when interpreting the results; captured in these survey responses are simply perspectives based on the meaning of these terms for each respondent. Moreover, within these perspectives, we did not examine individuals' impressions regarding the severity of stigma or the degree of perceived harm experienced in relation to narcissism, nor the depth of compassion for individuals suffering from narcissistic difficulties. Nevertheless, the findings provide a glimpse into how the public views and experiences people they perceive as narcissistic. Future research could probe deeper into these perspectives and engage public opinion more specifically regarding the impact of popular and social media messaging on narcissism. Further efforts could extend such work by examining the relationship between public attitudes and beliefs about narcissism and those held by clinicians and how these may impact help‐seeking among patients and the seeking of specialized training and provision of services among clinicians. Importantly, responses to the present survey indicate a role for public education about narcissistic dynamics and difficulties, as well as—and perhaps especially—a need for advocacy to reduce stigma and expand the availability and responsiveness of treatment. Hopefully the awareness regarding the negative interpersonal impact of pathological narcissism can be leveraged alongside public compassion to support improvement in clinical care.

## Funding

This research was supported by an award from Michael Smith Health Research BC, awarded to the first author, Scholar Award # 18317.

## Ethics Statement

This study was approved by the Behavioural Research Ethics Board of the University of British Columbia, and the procedures followed were in accordance with the 2013 revised Helsinki Declaration. All participants provided informed consent to participate in this study.

## Conflicts of Interest

The authors declare no conflicts of interest.

## Data Availability

The data that support the findings of this study are available from the corresponding author upon reasonable request.
